# Protocol: Strategy instruction for improving short‐ and long‐term writing performance on secondary and upper‐secondary students: A systematic review

**DOI:** 10.1002/cl2.1389

**Published:** 2024-03-03

**Authors:** André Kalmendal, Ida Henriksson, Thomas Nordström, Rickard Carlsson

**Affiliations:** ^1^ Department of Psychology Linnaeus University Växjö Sweden

## Abstract

This is the protocol for a Campbell systematic review. The objectives are as follows. This review aims to investigate the effectiveness of all types of teacher‐delivered classroom‐based strategy instruction aimed at students in the general population (all students) including struggling students (with or at‐risk of academic difficulties) in ages 12–19 for increasing writing performance. The majority of previous reviews scoped all outcomes presented in the primary studies. This review will solely focus on covering three most common outcomes: story quality, story elements and word count/length.

## 1 BACKGROUND

### 1.1 The problem, condition or issue

According to national assessments from several western countries such as the Netherlands, the USA, and the UK the results reveal poor results regarding students' writing proficiency (De Smedt & Van Keer, 2018; Inspectie van het Onderwijs, 2010; National Center for Education Statistics, 2012; Ofsted, 2000). The importance of writing in modern societies can not be overstated; even though we communicate in writing more than ever, students still have trouble with formal writing procedures such as planning, revising, and editing texts. The complexity of writing is well‐known and previous research shows that one promising way to enhance writing is through interventions consisting of self‐regulation training (Harris et al., 2003; Klein et al., 2021). In recent years, the method strategy instruction has received more attention which builds on self‐regulation as one of the core components. The consensus that self‐regulated learning is important for academic and lifelong learning is well established and Dignath and Veenman (2021) emphasize the role of direct Strategy Instruction as a framework for activating self‐regulated learning in the classroom. Strategy instruction is a collective name for interventions focusing on self‐regulation, self‐efficacy, and meta‐cognitive strategies. Olson and Land (2007) argue that we do “students a disservice when we offer a reductionist curriculum focusing primarily on skill and drill.” Instead of focusing on spelling and handwriting, students need to understand what it is that experienced writers do when they write. It is therefore crucial that students are introduced to cognitive strategies that underlie writing in a meaningful context. They argue that teachers should provide sustained and guided practices that can be internalized and performed independently by the students (Olson & Land, 2007). There are several types of strategy instruction conditions that utilize the concept of internalizing cognitive strategies to reach a higher level of writing. As of today, educational research using strategy instruction has been researched and tested on all grades in school. This review aims to investigate the effectiveness of strategy instruction on writing performance of all students, including struggling writers, in elementary school.

Previous literature (Dunn, 2012; Graham & Harris, 2005) shows that students with writing difficulties usually do not acquire (meta)‐cognitive strategies unless explicit and detailed information is provided by the teacher. Struggling writers usually spend less time in the pre‐writing phase such as reflecting on the topic, choosing the audience, and developing ideas, and instead start writing immediately after the assignment is given out. There are also students who have trouble getting started with writing, where students need help with writing by modeling texts. The idea of strategy instruction is to engage the students to actively create and understand the constructs of their personal strategies to develop self‐regulated learning (Chalk et al., 2005). Students who struggle with writing might therefore benefit even more from this intervention than typical students. Since having trouble writing can manifest itself in many ways (e.g., poor planning or having trouble getting started) it is reasonable to believe that strategy instruction is suitable as a whole‐class intervention for writers of different proficiency levels.

### 1.2 The intervention

Strategy Instruction is not a standardized manual‐based method but it typically addresses two core components of early‐stage writing development: discourse knowledge and self‐regulation (Kim, 2020), and can be described as a goal‐oriented mental activity often with an aim to solve a problem in writing within a learning situation (De Silva, 2015).

Discourse knowledge includes information on a specific genre of texts that the students are about to write, such as reports, speeches, and narrative or persuasive writing (Klein et al., 2021). To be self‐regulated learners, students need knowledge about the subject, the task, and the context in which they will apply their learning in order for them to reflect on their own learning processes (Woolfolk, 2021). The knowledge can be remembered using mnemonic strategies or more advanced maps of the key ideas.

**Figure 1:**
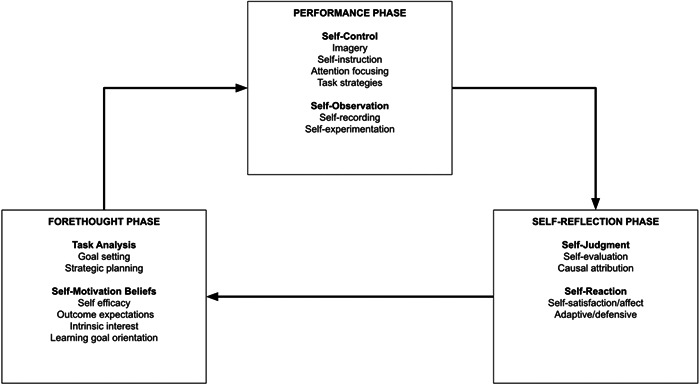
Phases and subprocesses of self‐regulation (Zimmerman, 2002).

Self‐regulation is the process we use to activate and sustain our thoughts, behaviors, and emotions to reach our goals (Perry & Rahim, 2011). According to Zimmerman (2002), self‐regulated learning is based on three phases: forethought, performance, and reflection (see Figure 1).

- Phase 1: forethought phase – The students need to set clear and reasonable goals and together with a plan to accomplish those goals. The students need motivation and high self‐efficacy to reach their goals.
- Phase 2: performance phase – In the performance phase the students must perform self‐control and learning strategies to stay engaged to the task. This might include mnemonics, imagery, attention focusing, and other techniques (Woolfolk, 2021). This stage also includes self‐observation in order for the students to understand how things are going and how they can change strategies if needed.
- Phase 3: reflection phase – The students now look back at their work and reflect and evaluate their performance. Questions like: What strategies worked? What did not work out? Were the goals reasonably set? are useful in order for the students to increase their self‐efficacy for the next similar task.

The concept of strategy instruction allows the creation of different interventions based on the two core components; discourse knowledge and self‐regulation, and the most researched intervention is Self‐Regulated Strategy Development which will be presented more thoroughly below followed by examples of other strategy instruction.

Self‐Regulated Strategy Development has been the most researched type of strategy instruction. This intervention addresses multiple processes of self‐regulation such as self‐instruction, self‐evaluation, goal‐setting, and self‐reinforcement (Klein et al., 2021). The Self‐Regulated Strategy Development intervention's primary focus lies in teaching students strategies for successfully completing an academic task by increasing knowledge and self‐regulatory procedures like goal setting, self‐instruction, and self‐monitoring (Harris et al., 2006). According to Graham and Harris (1993, 2005), Self‐Regulated Strategy Development also shows promising effects on students who are considered to be struggling with writing.

In Self‐Regulated Strategy Development, the lessons typically range from 20 to 60 min three times a week and the intervention is based on six stages: background knowledge, discussion, modeling, memorizing, supporting, and independent performance (Harris et al., 2003). The stages can be combined together and may take more than one lesson to complete. Below follows the Self‐Regulated Strategy Development stages of instruction as presented by Harris et al. (2013) and Graham and Harris (1993):

1. Background knowledge – In the first stage, students read and discuss works in the genre being addressed (e.g., reports, persuasive essays, etc.) to increase declarative, conditional, and procedural knowledge. Students may also be introduced to goal setting and self‐monitoring to develop self‐regulation.
2. Discussion – In this stage, it is important to establish students' commitment to learning strategies and to become collaborative partners. Now it is time to discuss the student's self‐regulation and writing abilities to learn more about the purpose, benefits, and how they can use them in their writing. Self‐monitoring (graphing) may be introduced to assist goal setting but might be skipped if the students are likely to negatively react to it.
3. Modeling – The teacher shows how the model is used and connects it to useful self‐instructions. Together, the teacher and the student discuss and analyze the strategies and the model performance and make changes if necessary, for example, a new mnemonic may be developed.
4. Memorizing – Start requiring and confirming memorization of strategies, mnemonics, and self‐instruction fitted for the students. The teacher makes sure that the students have memorized the strategies before independent performance takes place.
5. Supporting – Students and teachers together use writing and self‐regulation strategies to succeed in composing using strategy charts, self‐instruction sheets, and graphic organizers. Additional self‐regulation components used for managing the writing environment, use of imagery, and so forth, may be introduced. The criterion levels are increased gradually until the goals are met.
6. Independent performance – In the last stage, students should be able to use writing and self‐regulation strategies independently, with some support and monitoring by teachers if necessary. Plans for maintenance and generalization are discussed and implemented.
7. Background knowledge – In the first stage, students read and discuss works in the genre being addressed (e.g., reports, persuasive essays, etc.) to increase declarative, conditional, and procedural knowledge. Students may also be introduced to goal setting and self‐monitoring to develop self‐regulation.
8. Discussion – In this stage, it is important to establish students' commitment to learning strategies and to become collaborative partners. Now it is time to discuss the student's self‐regulation and writing abilities to learn more about the purpose, benefits, and how they can use them in their writing. Self‐monitoring (graphing) may be introduced to assist goal setting but might be skipped if the students are likely to negatively react to it.
9. Modeling – The teacher shows how the model is used and connects it to useful self‐instructions. Together, the teacher and the student discuss and analyze the strategies and the model performance and make changes if necessary, for example, a new mnemonic may be developed.
10. Memorizing – Start requiring and confirming memorization of strategies, mnemonics, and self‐instruction fitted for the students. The teacher makes sure that the students have memorized the strategies before independent performance takes place.
11. Supporting – Students and teachers together use writing and self‐regulation strategies to succeed in composing using strategy charts, self‐instruction sheets, and graphic organizers. Additional self‐regulation components used for managing the writing environment, use of imagery, and so forth, may be introduced. The criterion levels are increased gradually until the goals are met.
12. Independent performance – In the last stage, students should be able to use writing and self‐regulation strategies independently, with some support and monitoring by teachers if necessary. Plans for maintenance and generalization are discussed and implemented.

Cognitive Self‐Regulation Interventions aim to develop declarative knowledge about skills or procedures to a stage where the students can apply strategies to their own writing (Torrance et al., 2007). The intervention is adapted for use with typically developing students and therefore aims for less direct teacher oversight.

The cognitive Self‐Regulation Intervention program is built upon 10 weekly sessions with lessons lasting between 60 and 75 min as well as several homework tasks (Torrance et al., 2007). The sessions include interactive instructions in planning, setting rhetorical goals, generating the content, and developing a structure for the students' writing processes. Different mnemonics are used to remember the strategies and students are asked to emulate the teacher's modeled strategies in their homework. Feedback is given continuously by the teachers during the last three sessions and students are expected to write and produce their own list of self‐regulatory statements.

Tekster (Bouwer et al., 2018) is an example of a strategy instruction developed for Dutch students based on student's grades and level of writing proficiency, the focus is on teaching students a general writing strategy along with self‐regulation skills needed to use the strategy successfully. Tekster consists of three design principles: Writing strategies, Text structures, and Self‐regulation skills (Bouwer et al., 2018). The intervention includes a series of 16 lessons based on grade level. The lessons are typically between 45 and 60 min and aim to guide the students through all steps of the writing process. All three principles come with three modes of instruction: Observational learning, explicit instruction, and practice. The interactive learning activities include the first steps of observing and discussing, and applying different models of writing strategy at several stages of the writing process. Teachers introduce specific characteristics of text types through modeling, comparing texts, and explicit instructions. Further, the teacher introduces an assignment with a clear communicative goal and intended audience. Acronyms for the strategy are named and content is generated in keywords and gradually released into a text written using organized content. Students read each other texts and evaluate using questions and feedback resources. The last step of the intervention is revising the text based on the feedback received.

There will also be other interventions based on the concept of strategy instruction that includes goal‐setting, self‐evaluation, self‐instruction, self‐reinforcement, and so forth, and we aim to include all interventions in this review where authors explicitly use strategy instruction as a method for improving writing performance.

### 1.3 How the intervention might work

The idea of strategy instruction is to increase the student's discourse knowledge and self‐regulation in the domain of writing. A major purpose of the strategy is to deliver explicit instructions on the content in the genre the students are supposed to write within. Explicit instructions are one way to ease the processing demands associated with incorporating new procedures into an already heavily taxed cognitive system (Graham & Harris, 2005). Further, the students are treated as active collaborators in the learning process and the student's role and effort in learning the new strategies are emphasized and rewarded. The instructional components of the intervention aim to help the students to be creative, plan their writing, use a number of different writing strategies, and reinforce their own ideas to increase the level of their writing. The goal of the intervention is to enhance the students in all aspects of the writing process. A good writer has the knowledge and knows how to approach the writing process as a whole. This includes generating the content, organizing and creating a structure for the composition, formulating goals and plans, efficiently executing the mechanical aspects of writing, and revising and reformulating goals in the text (Chalk et al., 2005).

#### 1.3.1 Intervention in practice

Strategy instruction may be referred to as a constructivist approach to learning (Olson & Land, 2007). Together, the students and the teacher choose a strategy to solve a category of tasks and map it to a step‐by‐step plan in the classroom (Blik et al., 2016). The students progress throughout the stages as they fulfill the criteria in each stage, hence the instructions are rather criterion‐based than time‐based. The feedback and support are individualized by the instructor to adapt and be responsive to the student's needs.

Strategy instruction uses mnemonic devices to help students remember and apply different writing strategies. The mnemonic is usually adapted to the addressed language and might look like this: *TREE*: This strategy prompted students to *T*ell what you believe (State your topic sentence), give three or more *R*easons (Why do I believe this?), *E*nd it (Wrap it up right), and *E*xamine (Do I have all of my parts?) (Graham & Harris, 2005). Another example is *EKSTER* (which means magpie in Dutch), this strategy brings up several aspects of the writing process specific for Dutch students (Bouwer et al., 2018): *E*erst nadenken (think first), *K*iezen & ordenen (choose & organize), *S*chrijven (write), *T*eruglezen (reread), *E*valueren (evaluate), Reviseren (revise).

### 1.4 Why it is important to do this review

#### 1.4.1 Prior reviews

Several reviews have been conducted regarding strategy instruction (or similar interventions) and student performance over the past 10 years. In our search, we found 8 reviews (see Table 1) on the topic since 2011. We conducted a minor ROBIS check on the available reviews to evaluate the quality of the papers. We found that the previous reviews are of various quality and no review meets the requirement of a standardized systematic review (Higgins et al., 2022) and none were pre‐registered. Only one review (de Boer et al., 2018) provided information about long‐term effects, however, it was not solely focused on strategy instruction. The majority of the reviews do not differentiate between teacher‐delivered interventions in the classroom and external implementation of the intervention (e.g., by trained research staff outside of the classroom). Hence, they are conflating the efficacy and effectiveness of the intervention (Flay et al., 2005). Not separating between teacher and external implementation makes it harder to evaluate if the intervention works in applied authentic settings. Several of the previous reviews were conducted without any statistical analysis or standardized meta‐analytic methods (Finlayson & McCrudden, 2020; See & Gorard, 2020). Previous reviews also mixed reading and writing outcomes in the same analysis which makes the evidence hard to interpret (de Boer et al., 2018; Graham et al., 2018; Plonsky, 2011). The majority of the conducted reviews we found in this area did not use any Risk of Bias tools and had more in common with a scoping review rather than a meta‐analysis (Gillespie & Graham, 2014; de Boer et al., 2018; Donker et al., 2014). A summary of descriptives related to these eight reviews can be found in Table 1.

**Table 1:** Descriptives of previous literature.

Review	Population	Intervention	Comparison	Outcome	Included study design	Instructor	Pre‐reg.	Risk of bias
de Boer et al. (2018)	K‐12	Cognitive and meta‐cognitive strategies	Teaching as usual	Academic achievement	RCT	Teacher, researcher or computers	No	No
Donker et al. (2014)	K‐12	Cognitive and meta‐cognitive strategies	Teaching as usual	Student performance	RCT/QES	Not specified	No	No
Finlayson and McCrudden (2020)	K‐6	Writing instructions^a^	Not specified	Writing achievement	RCT/QES	Teacher implemented	No	No
Gillespie and Graham (2014)	1–12 with LD	Strategy instruction	Not specified	Writing quality	RCT/QES	Not specified	No	Quality score
Graham et al. (2012)	1–6	Strategy instruction	Not specified	Writing quality	RCT/QES	Not specified	No	No
Graham et al. (2018)	K‐12	Literacy programs^a^	Not specified	Writing and Reading	RCT	Teacher or researcher	No	No
Plonsky (2011)	K‐University	Cognitive and meta‐cognitive strategies	Teaching as usual	Strategy instruction effectiveness	QES	Not specified	No	No
See and Gorard (2020)	K‐12	Writing interventions^a^	Teaching as usual	Academic performance	RCT/QES/Longitudinal	Not specified	No	Quality assessment

Abbreviations: LD, learning disability; QES, Quasi‐Experimental study; RCT, randomized controlled trial.

^a^ Included interventions labeled as Strategy Instruction.

#### 1.4.2 Outcomes related to strategy instruction

Three common outcomes are primarily focused on in evaluations of strategy instruction: story quality, story elements or components, and length or word count (Harris et al., 2012; McKeown et al., 2018; Torrance et al., 2007; Gillespie & Graham, 2014; Chalk et al., 2005; Collins et al., 2021; Ennis, 2016; Graham & Harris, 2005; Klein et al., 2021; Sundeen, 2007). In assessing these outcomes it is common that students' manuscripts are transferred into a word document where spelling, punctuation, and capitalization errors are corrected to not bias the rater (Harris et al., 2012). This also removes the possibility of student identification by recognizing the style of handwriting.

It is common for studies to also include auxiliary measures that do not measure writing performance per se, but instead focus on different aspects of the intervention, such as adherence, preferences, whether they use the strategy, and how much time each student spends on different intervention stages. We will not assess these types of auxiliary measurements in this review; however, measures are of course interesting in understanding how an intervention works (e.g., whether students are using more meta‐cognitive strategies) and might be applicable for other types of research questions.

The three most common outcomes related to writing performance are presented below.

##### Story quality

Story quality or holistic quality comprises a measure of writing development by scoring a holistic measure of text quality. The outcome is typically rated on a rubric‐designed point‐scale, and the higher scores represent essays of better quality. The rubric design usually consists of 3–4 elements that vary depending on the topic of the essay, each element is separately scored and is then aggregated into one quality outcome. We will include story quality as an outcome as long as it is assessed quantitatively even if not scored as mentioned above. Some elements that can be seen in most articles are: Organization, Fluency, Development of support, Coherence, and Conventions. The raters vary in studies and include school teachers and special educators to researchers.

##### Story elements or components

Story elements or components are usually measured on a rubric scored point‐scale. Minimum and maximum scores vary between studies. Students get points by including different elements or components such as: topic sentences, supporting details, transition words, explanations, and endings (e.g., a student can get 0 points if the element is missing, 1 point if the element was included, and 2 points if the element was elaborated or highly developed). If the outcomes are graded in a different way than a rubric scored point‐scale, we aim to include the outcome as long as it is measured quantitatively. As in story quality, the rates vary between studies and include teachers, special educators, and researchers.

##### Length and word count

Length and word count are measured as raw scores that represent the total number of words written in the essay without regard to grammatical accuracy or spelling.

##### Long‐ and short‐term measurements

According to the Swedish Agency for Health Technology Assessment and Assessment of Social Services (Statens beredning för medicinsk och social utvärdering [SBU] [The Swedish Council on Health Technology Assessment], 2014), there is no standardized time for a follow‐up measurement in studies of literacy. Although, we argue that a 6‐month period follow‐up time would be interesting for a teacher because it would also provide information regarding if the intervention still shows effects going into the next semester.

#### 1.4.3 The contribution of this review

The study aims to review evidence‐based research for strategy instruction interventions to improve student performance in writing. This includes all students in the classrooms as well as students who are struggling with writing. We focus on students ages 12–19, as this is the critical period in writing development where strategy instruction can be expected to be most beneficial. At the age of 12 years, most students will already have mastered the basics of how to write (e.g., spelling, basic grammar), and writing is used as a tool for learning and demonstrating knowledge. Thus, the skills needed to start producing longer and more advanced texts becomes an essential part of students' academic success.

In line with the Salamanca declaration, the field of education has made historical changes toward more inclusive teaching but there are challenges in delivering classroom‐based interventions that target all students, including struggling academics (Shaw & Pecsi, 2021). One important aspect of educational research is therefore to provide teachers with evidence‐based guidelines for teaching writing in daily practice that targets all students in the classroom (De Smedt & Van Keer, 2018; UNESCO, 2014).

## 2 OBJECTIVES

This review aims to investigate the effectiveness of all types of teacher‐delivered classroom‐based strategy instruction aimed at students in the general population (all students) including struggling students (with or at‐risk of academic difficulties) in ages 12–19 for increasing writing performance. The majority of previous reviews scoped all outcomes presented in the primary studies. This review will solely focus on covering three most common outcomes: story quality, story elements and word count/length.

### 2.1 Primary research questions

What are the short‐term effects of teacher‐delivered strategy instruction on all students (ages 12–19) writing performance when compared to teaching as usual?

What are the short‐term effects of teacher‐delivered strategy instruction on struggling writers' (ages 12–19) writing performance when compared to teaching as usual?

### 2.2 Secondary research questions

The review will also include the following secondary research questions. We consider them secondary as we are unsure whether there is sufficient research in this area to answer them.

What are the long‐term effects of teacher‐delivered strategy instruction on all students' (12−19) writing performance when compared to teaching as usual?

What are the long‐term effects of teacher‐delivered strategy instruction on struggling writers (12–19) writing performance when compared to teaching as usual?

### 2.3 Moderator analysis

We will conduct a subgroup analysis for the different types of strategy instruction (SRSD, CSRI, Tekster, Other).

## 3 METHODS

### 3.1 Criteria for considering studies for this review

#### 3.1.1 Types of studies

- Studies must have a randomized controlled trials (RCTs) design, and/or cluster RCTs design. We will also include quasi‐experimental designs, and/or cluster quasi‐experimental designs, which use both control groups and pretests.

Controls should be carefully matched (e.g., same‐year students, same or similar school) with demonstrated baseline equivalence. Single group pre‐post comparisons are excluded as well as studies that compare with norm data or similar types of statistical controls.

We want the review to be as comprehensive as possible and therefore include both (cluster) RCTs and quasi‐experimental studies. In educational research, it is hard to conduct blinded RCTs (e.g., parents or teachers unaware that the control group students did not receive an intervention).

#### 3.1.2 Types of participants

- Studies should include students (age 12–19) attending regular, private, or public, schools in grades 7–12 in typical classroom settings in OECD countries.

The population eligible for the review includes all students (age 12–19) attending regular, private, or public, schools in grades 7–12. We will only include students in typical classroom settings and not focus on classes in special schools. Students attending special schools, reading clinics, or equivalent are thus not included in this study and should be focused on in another systematic review because it warrants other approaches than ours.

To make the selected studies comparable across included studies, we will only include studies carried out in OECD countries due to the similarity in school settings and teaching as usual conditions.

An important sub‐population of interest for our secondary research question in this review is students who struggle with writing. We will include all struggling writers regardless of their causes (i.e., different learning difficulties or disabilities) if they are participating in regular classrooms. Because the field lacks agreed‐upon criteria for who is considered a struggling writer (Dunn, 2012), we will include students who have documented difficulties with writing (e.g., details provided by the school or screened by the researchers) and perform on or below the 25th percentile in normed‐referenced tests (a typical cutoff for struggling readers, which is fairly comparable) in at least one of the following areas: vocabulary, spelling, sentence combination or story composition, such as provided by the standardized writing test TOWL, Comprehensive Receptive and Expressive Vocabulary Test, Receptive One‐Word Picture Vocabulary Test‐Revised, or similar.

#### 3.1.3 Types of interventions

- Studies should have teacher‐delivered classroom‐based explicitly stated strategy instruction intervention for improving writing performance.

To be included, studies should focus on teacher‐delivered classroom‐based strategy instruction for improving writing performance. Strategy Instruction includes specific use of strategies that involve students planning, writing, revising, and/or editing texts (Gillespie & Graham, 2014). There may be other interventions based on the concept of Strategy Instruction that includes goal‐setting, planning, revising, and so forth, and we aim to include all interventions.

The comparison/control group will be teaching as usual, which does not include strategy instruction or another subtype of strategy instruction. We will code what the comparison/control group does to the extent possible when information is given by the authors.

The intervention is restricted to implementations in classroom settings, during the regular school year.

#### 3.1.4 Types of outcome measures

##### Primary outcomes

- Studies should provide at least one of the following outcomes: story quality, story elements or components, and length or word count.

The outcomes included will be writing performance with both short and follow‐up measurement points. Writing performance is typically systematically assessed and/or scored using standardized tests (Finlayson & McCrudden et al., 2020; Harris et al., 2006; Torrance et. al., 2007).

For the short‐term outcomes, we will use measures that are collected immediately after the intervention is finished. For long‐term outcomes, a minimum of 6 months after the interventions is required to be regarded as long‐term in this study.

##### Secondary outcomes

There are no planned secondary outcomes for this review. If we find other outcomes than our core outcomes, we will present them in a table but not include them in our main analysis. However, if we find another outcome that exists in the majority of our selected studies, we will include it in our analysis.

#### 3.1.5 Types of settings

- Studies that focus on students attending special schools, reading clinics, or equivalent, as well as summer schools and after‐school programs, will be excluded.
- Studies with an active control group (e.g., other writing interventions) will be excluded.

### 3.2 Search methods for identification of studies

The search strategy is developed with recommendations based on the Campbell guide provided by Kugley et al. (2017). Our strategy to find relevant studies will be described below, additional information about the search can be found in Supporting Information: Appendix A.

#### 3.2.1 Limitations and restrictions

The Self‐Regulation Strategy Development intervention was developed in 1992 (Graham & Harris, 1993) therefore, all searches will be restricted to publications after 1990.

Due to the nature of our language limitation, we will only select studies written in English. In alignment with the Cochrane handbook, our search will be updated within 6 months of publication (Higgins et al., 2022).

#### 3.2.2 Filters

As stated in Kugley et al. (2017), predefined filters should be used with caution outside of medical and health sciences. However, to get studies with specific designs we will use the recommended strategies for finding methodologically sound studies in each database (e.g., strategies developed by Cochrane specifically for PsycInfo, https://work.cochrane.org/psycinfo).

### 3.3 Electronic searches

#### 3.3.1 Databases

We will search different databases through Linnaeus University access. Potentially relevant studies will be identified through these databases:

- APA PsycInfo (ProQuest)
- ERIC (ProQuest)
- Linguistics and Language Behavior Abstracts (ProQuest)
- MLA International Bibliography (EBSCO)
- Web of Science: Core collection (Clarivate)
  - SCI‐Expanded (1900–present)
  - SSCI – (1955–present)
  - AHCI – (1975–present)
  - ESCI – (2005–present)

#### 3.3.2 Search terms

The search term provided below is tailored toward PsycInfo. A report of the search string will be presented in Supporting Information: Appendix A. The search is divided into the categories: Study type, Intervention, and Population. Our preliminary searches in PsycINFO resulted in 1444 hits without filtering for peer‐reviewed articles (date: 20230428).

(MAINSUBJECT.EXACT(“High School Education”) OR MAINSUBJECT.EXACT(“High School Students”) OR MAINSUBJECT.EXACT(“High Schools”) OR MAINSUBJECT.EXACT(“Junior High School Students”) OR MAINSUBJECT.EXACT(“Junior High Schools”) OR MAINSUBJECT.EXACT(“Middle School Education”) OR MAINSUBJECT.EXACT(“Middle School Students”) OR MAINSUBJECT.EXACT(“Middle Schools”) OR MAINSUBJECT.EXACT(“Secondary Education”) OR ti,ab,if(“grade* 7” OR “grade* seven” OR “seventh grade”) OR ti,ab,if(“grade* 8” OR “grade* eight” OR “eighth grade”) OR ti,ab,if(“grade* 9” OR “grade* nine” OR “ninth grade”) OR ti,ab,if(“grade* 10” OR “grade* ten” OR “tenth grade”) OR ti,ab,if(“grade* 11” OR “grade* eleven” OR “eleventh grade”) OR ti,ab,if(“grade* 12” OR “grade* twelve” OR “twelfth grade”) OR ti,ab,if(gymnasium*) OR ti,ab,if(“high school*”) OR ti,ab,if(“highschool*”) OR ti,ab,if(“juniorhigh”) OR ti,ab,if(K‐12) OR ti,ab,if(K12) OR ti,ab,if(“lower secondary”) OR ti,ab,if(“middle” PRE/1 school*) OR ti,ab,if(pupil OR pupils) OR ti,ab,if(“secondary” PRE/1 (school* OR education* OR level OR grade)) OR ti,ab,if(student OR students) OR ti,ab,if(“upper secondary”)) AND (MAINSUBJECT.EXACT(“Literacy”) OR MAINSUBJECT.EXACT(“Literacy Programs”) OR MAINSUBJECT.EXACT(“Writing Skills”) OR MAINSUBJECT.EXACT.EXPLODE(“Written Communication”) OR mainsubject.Exact(“writing”) OR ti,ab,if(“writing”)) AND (MAINSUBJECT.EXACT(“Educational Objectives”) OR MAINSUBJECT.EXACT(“Goals”) OR MAINSUBJECT.EXACT(“Goal Orientation”) OR MAINSUBJECT.EXACT(“Goal Setting”) OR MAINSUBJECT.EXACT(“Individualized Instruction”) OR MAINSUBJECT.EXACT(“Learning Strategies”) OR MAINSUBJECT.EXACT(“Metacognition”) OR MAINSUBJECT.EXACT(“Mnemonic Learning”) OR MAINSUBJECT.EXACT(“Self‐Efficacy”) OR MAINSUBJECT.EXACT(“Self‐Evaluation”) OR MAINSUBJECT.EXACT(“Self‐Monitoring”) OR MAINSUBJECT.EXACT(“Self‐Regulated Learning”) OR MAINSUBJECT.EXACT(“Self‐Regulation”) OR MAINSUBJECT.EXACT(“Strategies”) OR ti,ab,if((Goal OR goals) NEAR/1 setting) OR ti,ab,if(“learning skill”) OR ti,ab,if(“learning skills”) OR ti,ab,if(“learning strat*”) OR ti,ab,if(metacognit*) OR ti,ab,if(meta‐cognit*) OR ti,ab,if(Mnemonic OR Mnemonics) OR ti,ab,if(self‐instruction) OR ti,ab,if(self‐instructions) OR ti,ab,if(self‐instructional) OR ti,ab,if(self‐evaluat*) OR ti,ab,if(self‐monitor*) OR ti,ab,if(selfmonitor*) OR ti,ab,if(self PRE/1 effic*) OR ti,ab,if(selfeffic*) OR ti,ab,if(self‐regulat*) OR ti,ab,if(selfregulat*) OR ti,ab,if(“strat* instruction*”) OR ti,ab,if(“strat*use”) OR ti,ab,if(“study skill”) OR ti,ab,if(“study skills”) OR ti,ab,if(“study strat*”)) AND (ti,ab,su(experiment*) OR ti,ab,su(“group”) OR ti,ab,su(“groups”) OR ti,ab,su(intervention*) OR ti,ab,su(random NEAR/1 (distributed OR assigned OR sampling)) OR ti,ab,su(randomized) OR ti,ab,su(randomly) OR ti,ab,su(“trial”) OR ti,ab,su(“trials”) OR ti,ab,su(quasi*) OR ti,ab,su(quasiexperiment*)).

### 3.4 Searching other resources

#### 3.4.1 Hand search

A hand search will be performed on the editions released from 2018 to 2023 to capture relevant studies recently published that might not have been indexed in any databases. The following selected journals had a high frequency of publishing relevant literature capturing strategy instruction and writing performance.

- Intervention in School and Clinic
- Reading and writing quarterly
- Journal of Learning Disabilities Quarterly

#### 3.4.2 Gray literature

##### Search for working papers/conference proceedings

To identify relevant conference papers, academic clearinghouses and repositories for working papers additional searches will also be conducted within a timeframe of the past 10 years (2013–2023). Due to the nature of our research question, it is unlikely that government documents provide studies with experimental research designs, therefore, none will be sought.

- European Educational Research Association
- American Educational Research Association
- What Works Clearinghouse
- Campbell Collaboration
- Education Endowment Foundation

##### Search for dissertations

The following databases selected in our electronic search index dissertations and theses in their catalog:

- APA PsycInfo (ProQuest)
- ERIC (ProQuest)

Additional searches in national repositories are also conducted to find unpublished or non peer‐review content:

- DIVA – Swedish repository for research publications and theses
- EThOS – British repository for doctoral theses
- NDLTD – Networked Digital Library of Theses and Dissertations
- OATD – Open Access Theses and Dissertations

##### Citation searching

To identify both published studies and gray literature we will use forward and backward citation‐tracking strategies. The primary strategy is to cite‐track related systematic reviews and meta‐analyses. We also plan to check the citations and reference list of included primary studies for new leads.

##### Contacts to international experts

Based on our initial screening of existing meta‐analyses mentioned in previous literature, we plan to contact the following international expert in the related area to identify unpublished and ongoing studies:

- Steve Graham
- Karen R. Harris
- Radhika De Silva
- Mark Torrance

### 3.5 Data collection and analysis

#### 3.5.1 Selection of studies

The screening procedure will be conducted independently in Rayyan (Ouzzani et al., 2016) by at least two reviewers from the systematic review team. A third reviewer will help resolve eventual conflicts. Our search and screening procedure will be presented in a flow diagram in accordance with PRISMA (Page et al., 2021).

The screening process will be divided into two stages. The first stage is made based on titles and abstracts and the second stage decision is made out of full‐text reads. In the title and abstract reading stage, a study will be excluded if one answer to questions 1–6 in Supporting Information: Appendix B is “No.” If the answer to these questions is “Yes” or “Uncertain” it will be moved forward to full text read in the second stage. These questions are based on our PICOS.

In the full‐text screening, each report will be screened again against the first six questions, as well as three additional ones. A study will only be included in the review if the answers to all questions are “Yes.” If a study receives the answer “Uncertain” or if there is a disagreement regarding the eligibility in the full‐text screening stage it will be resolved by the review authors. If deemed necessary, the study author/s will be contacted to provide more information.

#### 3.5.2 Data extraction and management

Two review authors will code independently and extract data from included studies. Any disagreement will be resolved through discussion. The data that will be extracted is bibliographical data, the characteristics of participants, sample size, intervention, control group, research design, and outcomes. All data will be coded using Google sheets and stored accordingly on Google Drive and the extracted data will also be uploaded to the Open Science Framework. Our codebook is presented in Supporting Information: Appendix C. Before starting the data extraction for the review, we are going to pilot our code book on five articles.

In cases where studies have multiple assessors (e.g., two independent raters), the aggregated score will be extracted. If studies contain standardized test scores on an aggregated level (e.g., both reading and spelling combined) and we are not able to extract the writing outcome exclusively the study will be excluded.

If a study contains a broader PICOS, for example, students in grades K‐12, only the subset of the sample that is eligible for our study will be extracted. If we can not extract the eligible subset, the data will be considered missing, and the study will not be included in this review.

#### 3.5.3 Assessment of risk of bias in included studies

As recommended by Cochrane when assessing randomized trials, RoB‐2 will be used to assert the risk of bias (Sterne et al., 2019). Two reviewers from the research group will independently review the risk of bias, any disagreement will be resolved between the two reviewers. We will be using the original version as well as the test version for cluster‐randomized trials when needed. This tool includes five domains that cover all types of bias that are currently understood to affect the results:

1. Bias arising from the randomization process
2. Bias due to deviations from intended interventions
3. Bias due to missing outcome data
4. Bias in the measurement of the outcome
5. Bias in the selection of the reported result

For the Quasi‐experimental studies, we will be using the tool ROBINS‐I (Sterne et al., 2016). This tool is similar to RoB‐2 but includes seven domains:

1. Bias due to confounding
2. Bias in the selection of participants for the study
3. Bias in the classification of interventions
4. Bias due to deviations from intended interventions
5. Bias due to missing data
6. Bias in the measurement of outcomes
7. Bias in the selection of the reported results

The response options for each question regarding bias consists of Yes, probably yes, probably no, No, and No information. In RoB‐2, the results add up to a domain‐level judgment about the risk of bias which includes the levels of low‐risk of bias, some concerns, and High risk of bias. In ROBINS‐I, the results add up to low risk, moderate risk, serious risk, critical risk of bias. We do not expect to find too many studies with a low‐risk of bias therefore, we will conduct our main analysis on low‐ and moderate‐risk of bias. However, a sensitivity analysis will be performed including the studies with a serious risk in ROBINS‐I and a high‐risk of bias in RoB‐2. Any primary study assessed with critical risk of bias will be removed from the analysis in line with provided recommendations (Sterne et al., 2016).

#### 3.5.4 Measures of treatment effect

Based on our initial screening of the literature, we expect all core outcomes to be continuous variables reported as means and standard deviations for the pre‐test, post‐test, and, for some studies, follow‐up(s). We will extract one single measure of the means and standard deviations for each core outcome, both aggregated across all students (i.e., the main reporting of all studies), normal performing students (for studies that report this), and for the sub‐group of struggling writers separately (for studies that report this). If several assessments for the same core outcome are presented we will extract the focal test, if a focal test cannot be determined, one random selected test score will be extracted. The reason for this is to remove any dependency that can occur when extracting several effect sizes for the same outcome (Hedges et al., 2010).

Next, we will calculate the Standardized Means Differences (SMD) for the comparison between the control and intervention group for the post‐test, as well as for any follow‐up test, using the escalc function in metafor (Viechtbauer, 2010), with the measure argument SMD (i.e., Hedge's *g*, metafor, Viechtbauer, 2010). The effect sizes will be coded in a way that positive effects are favoring the intervention group. The full R‐code can be found in Supporting Information: Appendix D.

#### 3.5.5 Unit of analysis issues

Because the interventions included in this review are always conducted in the regular classroom setting, the studies will most likely be clustered randomized trials or clustered quasi‐experimental trials. However, it might vary whether the clustering is on the level of classrooms or schools. According to the Cochrane handbook, the ideal information to extract from a cluster‐randomized trial is the direct estimate of the required measure from an appropriate analysis, such as Multilevel modeling or Generalized Estimating Equations. However, there is currently no explicit standard in how this is reported in the literature, meaning that we cannot expect to find comparable metrics to extract (e.g., studies might use different standardizers when calculating effect sizes from multilevel‐models). Instead, we will use the alternative approach of adjusting for the design effect (Higgins et al., 2008). The main idea is to correct the sample size of each clustered trial, by dividing the sample size by the design effect. The design effect is calculated as: 1 + (M − 1) × ICC; where M = average cluster size and ICC = intracluster correlation coefficient (Hedges, 2007).

In cases where ICC is not reported, we will use an external ICC. The closest estimates we could find are provided by Hedges and Hedberg (2007) and are based on reading performance at the school level. The ICC varies quite a bit between different school grades, and there's no exact figure that matches grades 7–12, but based on the range of ICCs we find 0.1 to be reasonable, albeit rough, estimate to use as our default as it also is consistent with the findings of Ahn et al. (2012). We will use the same estimate for classroom clusters as well. We will also conduct sensitivity analyses based on assumed ICC of 0.05 and 0.2.

In cases where a study consists of several groups that use different types of interventions using strategy instruction, we will analyze these groups separately.

#### 3.5.6 Dealing with missing data

If the selected studies do not provide the statistics (M and SD) to estimate the effect size, or some other information that's part of our code sheet (e.g., the country it was conducted in), we will request the missing information from the authors of the primary studies. If we still cannot obtain the information, the study will be included in the review but excluded from the analysis that needs that information (e.g., missing outcomes means it's excluded from the meta‐analytical synthesis).

#### 3.5.7 Assessment of heterogeneity

The heterogeneity will be assessed based on the random‐effects model (in metafor, see Supporting Information: Appendix D for the full code). In the random‐effects model, the studies are assumed to be sampled from a larger population of possible studies. This distribution is assumed to be normally distributed with the variance of *τ* ^2^, that is, *N*(*µ*, *τ* ^2^). Hence, *τ* ^2^ is a direct estimate of the heterogeneity. However, that also means that *τ* is the standard deviation of this population of possible studies, which is, in our opinion, easier to interpret as it is on the same metric as the outcome (SMD). For example, if the SMD is 0.5 with a *τ* of 0.25, we know that on average, the heterogeneity is half as large as the effect itself. We will also calculate *I* ^2^ statistic, as it can be interesting to understand how much of the variance across studies is due to heterogeneity and how much is due to sampling variance.

#### 3.5.8 Assessment of reporting biases

To examine possible publication bias, we will use a contour‐enhanced funnel plot, Eggers' test (Egger et al., 1997) as well as PET‐PEESE (Stanley & Doucouliagos, 2014) to test for and adjust for small‐study bias (which is often, but not always, due to publication bias). We will also use 3PSM (Iyengar & Greenhouse, 1988), which in contrast is a selection model (Hedges & Vevea, 1996; Hedges, 1984; Vevea & Woods, 2005) that adjusts the weight of different studies (i.e., lower weight for statistically significant studies) to adjust for that non‐significant studies might be missing in the literature.

Simulation studies made by Carter et al. (2019), Stanley (2017), and McShane et al. (2016), suggest that these publication bias methods require a large number of studies (e.g., *K* > 20) to perform well in terms of accuracy of adjustment and power. We will thus not apply these methods (except the funnel plot) if *K* < 10, and still remain cautious about the result if *K* < 20. It might be tempting to resolve this by pooling across both types of designs. However, we argue that would be a mistake, as it would risk confounding small‐sample bias with design, as RCT studies usually have smaller sample sizes, and are also arguably more likely to be published regardless of whether results are statistically significant or not. Further, that would also mean that we would estimate an effect size that does not properly match our research questions.

#### 3.5.9 Data synthesis

We will conduct a separate analysis for RCT and quasi‐experimental designs to determine the effects of each study design. As we expect heterogeneity in the underlying effect sizes that are studied across studies (e.g., due to different class sizes, grades, and resources), our main synthesis will be a random‐effects meta‐analysis (Borenstein et al., 2009). All effect sizes will be presented as Hedges g due to the nature of us expecting a low amount of studies included (N < 20). Hedges g uses pooled standard deviation to adjust for small sample sizes (Borenstein et al., 2009).

$${\mathrm{Hedges}}^{\prime }g=\frac{M1-M2}{\text{SDpooled}},$$

where:

M1 − M2 = difference in means,

SDpooled = pooled and weighted standard deviations.

We will use the Restricted maximum likelihood estimator, and our alpha level will be set to 0.05. The results will also be illustrated in a forest plot along with the 95% confidence intervals. We will conduct the meta‐analysis in the R‐package metafor (Viechtbauer, 2010). The full code can be found in Supporting Information: Appendix D.

#### 3.5.10 Subgroup analysis and investigation of heterogeneity

There will be a separate contrast analysis for all students, typically developing students, as well as struggling writers.

For our moderator analysis question, we aim to conduct a subgroup analysis of the different types of interventions. However, we consider this exploratory since the analysis is dependent on how many studies use a specific method, and we'll have to decide in a later stage on what type of comparison (if any) is meaningful.

#### 3.5.11 Sensitivity analysis

We plan to conduct a summary table for our sensitivity analyses including the analyses mentioned above; the SMD with ICC levels of 0.10 and 0.15; and analysis with studies assigned with a high‐risk of bias. We will also perform a leave‐one‐out analysis to see whether the estimated results are robust to the influence of a single study.

#### 3.5.12 Summary of findings and assessment of the certainty of the evidence

A summary of findings will be presented in the review including:

- Population
- Setting
- Intervention
- Comparison
- All core outcomes for all students (% improvement)
- All core outcomes for struggling writers (% improvement)
- Number of participants
- Bias Check overview: RoB2 or ROBINS‐I
- Comments
- Explanations

## CONTRIBUTIONS OF AUTHORS

- Content: André Kalmendal
- Systematic review methods: André Kalmendal, Thomas Nordström, Rickard Carlsson
- Statistical analysis: André Kalmendal, Rickard Carlsson
- Information retrieval: Ida Henriksson

## DECLARATIONS OF INTEREST

No conflict of interest.

### Preliminary timeframe

Approximate date for submission of the systematic review: May 2025.

## Supporting information

Supporting information.

Supporting information.

Supporting information.

Supporting information.
