# Supplementary material for: Protocol: Strategy instruction for improving short‐ and long‐term writing performance on secondary and upper‐secondary students: A systematic review
Source: Campbell Syst Rev. 2024 Mar 3;20(2):e1389. doi: 10.1002/cl2.1389 (PMC10909389; doi:10.1002/cl2.1389)
Supplement: Supplementary file 1 — Supporting information. [file CL2-20-e1389-s001.docx]

Appendix A - Search terms

| Set# | Searched for | Databases | Results |
| --- | --- | --- | --- |
| S1 | ti,ab,su(groups) | APA PsycInfo® | 1071356 |
| S2 | ti,ab,su(experiment*) | APA PsycInfo® | 656816 |
| S3 | ti,ab,su(quasi*) | APA PsycInfo® | 19511 |
| S4 | ti,ab,su(intervention*) | APA PsycInfo® | 464334 |
| S6 | ti,ab,su(randomized) | APA PsycInfo® | 99036 |
| S7 | ti,ab,su(randomly) | APA PsycInfo® | 78639 |
| S8 | ti,ab,su(Trial) | APA PsycInfo® | 217878 |
| S9 | ti,ab,su(Random NEAR/1 (distributed OR assigned)) | APA PsycInfo® | 422 |
| S10 | ti,ab,su(groups) OR ti,ab,su(experiment*) OR ti,ab,su(quasi*) OR ti,ab,su(intervention*) OR ti,ab,su(randomized) OR ti,ab,su(randomly) OR ti,ab,su(Trial) OR ti,ab,su(Random NEAR/1 (distributed OR assigned)) | APA PsycInfo®  These databases are searched for part of your query. | 2002919 |
| S11 | noft(writ*) | APA PsycInfo® | 169110 |
| S12 | noft(perform*) | APA PsycInfo® | 675005 |
| S13 | noft(writ*) OR noft(perform*) | APA PsycInfo®  These databases are searched for part of your query. | 825228 |
| S14 | noft(self* NEAR/1 Effic*) | APA PsycInfo® | 55789 |
| S15 | noft("study strat*") | APA PsycInfo® | 1066 |
| S16 | noft("learning skill*") | APA PsycInfo® | 1648 |
| S17 | noft("study skill*") | APA PsycInfo® | 1569 |
| S18 | noft("strat* use") | APA PsycInfo® | 4106 |
| S19 | noft("strat* instruction") | APA PsycInfo® | 1140 |
| S20 | noft(self-regul*) | APA PsycInfo® | 28018 |
| S21 | noft(metacognit*) | APA PsycInfo® | 14192 |
| S22 | noft("learning strat*") | APA PsycInfo® | 14322 |
| S23 | noft(Goal* NEAR/1 setting) | APA PsycInfo® | 9462 |
| S24 | noft(self* NEAR/1 Effic*) OR noft("study strat*") OR noft("learning skill*") OR noft("study skill*") OR noft("strat* use") OR noft("strat* instruction") OR noft(self-regul*) OR noft(metacognit*) OR noft("learning strat*") OR noft(Goal* NEAR/1 setting) | APA PsycInfo®  These databases are searched for part of your query. | 118999 |
| S25 | noft(secondary NEAR/1 school*) | APA PsycInfo® | 24424 |
| S26 | noft("upper secondary" NEAR/1 school*) | APA PsycInfo® | 664 |
| S27 | noft(high NEAR/2 School*) | APA PsycInfo® | 111045 |
| S28 | noft(Junior NEAR/2 School*) | APA PsycInfo® | 18515 |
| S29 | noft(Middle NEAR/1 School*) | APA PsycInfo® | 24212 |
| S30 | noft("secondary education") | APA PsycInfo® | 11510 |
| S31 | ti,ab(student*) | APA PsycInfo® | 542422 |
| S32 | ti,ab(pupil*) | APA PsycInfo® | 27317 |
| S33 | noft(secondary NEAR/1 school*) OR noft("upper secondary" NEAR/1 school*) OR noft(high NEAR/2 School*) OR noft(Junior NEAR/2 School*) OR noft(Middle NEAR/1 School*) OR noft("secondary education") OR ti,ab(student*) OR ti,ab(pupil*) | APA PsycInfo®  These databases are searched for part of your query. | 614416 |
| **S34** | (noft(secondary NEAR/1 school*) OR noft("upper secondary" NEAR/1 school*) OR noft(high NEAR/2 School*) OR noft(Junior NEAR/2 School*) OR noft(Middle NEAR/1 School*) OR noft("secondary education") OR ti,ab(student*) OR ti,ab(pupil*)) AND (noft(self* NEAR/1 Effic*) OR noft("study strat*") OR noft("learning skill*") OR noft("study skill*") OR noft("strat* use") OR noft("strat* instruction") OR noft(self-regul*) OR noft(metacognit*) OR noft("learning strat*") OR noft(Goal* NEAR/1 setting)) AND (noft(writ*) OR noft(perform*)) AND (ti,ab,su(groups) OR ti,ab,su(experiment*) OR ti,ab,su(quasi*) OR ti,ab,su(intervention*) OR ti,ab,su(randomized) OR ti,ab,su(randomly) OR ti,ab,su(Trial) OR ti,ab,su(Random NEAR/1 (distributed OR assigned))) | APA PsycInfo®  These databases are searched for part of your query.    **Results without publication date and peer review.** | 5908 |
| **S35** | (noft(secondary NEAR/1 school*) OR noft("upper secondary" NEAR/1 school*) OR noft(high NEAR/2 School*) OR noft(Junior NEAR/2 School*) OR noft(Middle NEAR/1 School*) OR noft("secondary education") OR ti,ab(student*) OR ti,ab(pupil*)) AND (noft(self* NEAR/1 Effic*) OR noft("study strat*") OR noft("learning skill*") OR noft("study skill*") OR noft("strat* use") OR noft("strat* instruction") OR noft(self-regul*) OR noft(metacognit*) OR noft("learning strat*") OR noft(Goal* NEAR/1 setting)) AND (noft(writ*) OR noft(perform*)) AND (ti,ab,su(groups) OR ti,ab,su(experiment*) OR ti,ab,su(quasi*) OR ti,ab,su(intervention*) OR ti,ab,su(randomized) OR ti,ab,su(randomly) OR ti,ab,su(Trial) OR ti,ab,su(Random NEAR/1 (distributed OR assigned))) AND pd(19920101-20220201) | APA PsycInfo®  These databases are searched for part of your query.    **Results with publication date** | 5589 |
| S36 | (noft(secondary NEAR/1 school*) OR noft("upper secondary" NEAR/1 school*) OR noft(high NEAR/2 School*) OR noft(Junior NEAR/2 School*) OR noft(Middle NEAR/1 School*) OR noft("secondary education") OR ti,ab(student*) OR ti,ab(pupil*)) AND (noft(self* NEAR/1 Effic*) OR noft("study strat*") OR noft("learning skill*") OR noft("study skill*") OR noft("strat* use") OR noft("strat* instruction") OR noft(self-regul*) OR noft(metacognit*) OR noft("learning strat*") OR noft(Goal* NEAR/1 setting)) AND (noft(writ*) OR noft(perform*)) AND (ti,ab,su(groups) OR ti,ab,su(experiment*) OR ti,ab,su(quasi*) OR ti,ab,su(intervention*) OR ti,ab,su(randomized) OR ti,ab,su(randomly) OR ti,ab,su(Trial) OR ti,ab,su(Random NEAR/1 (distributed OR assigned))) AND (pd(19920101-20220201) AND PEER(yes)) | APA PsycInfo®  These databases are searched for part of your query.    **Results with publication date and peer review** | 3340 |

Search string with publication date:

(noft(secondary NEAR/1 school*) OR noft("upper secondary" NEAR/1 school*) OR noft(high NEAR/2 School*) OR noft(Junior NEAR/2 School*) OR noft(Middle NEAR/1 School*) OR noft("secondary education") OR ti,ab(student*) OR ti,ab(pupil*)) AND (noft(self* NEAR/1 Effic*) OR noft("study strat*") OR noft("learning skill*") OR noft("study skill*") OR noft("strat* use") OR noft("strat* instruction") OR noft(self-regul*) OR noft(metacognit*) OR noft("learning strat*") OR noft(Goal* NEAR/1 setting)) AND (noft(writ*) OR noft(perform*)) AND (ti,ab,su(groups) OR ti,ab,su(experiment*) OR ti,ab,su(quasi*) OR ti,ab,su(intervention*) OR ti,ab,su(randomized) OR ti,ab,su(randomly) OR ti,ab,su(Trial) OR ti,ab,su(Random NEAR/1 (distributed OR assigned))) AND pd(19920101-20220201)
