# Supplementary material for: Protocol: Strategy instruction for improving short‐ and long‐term writing performance on secondary and upper‐secondary students: A systematic review
Source: Campbell Syst Rev. 2024 Mar 3;20(2):e1389. doi: 10.1002/cl2.1389 (PMC10909389; doi:10.1002/cl2.1389)
Supplement: Supplementary file 2 — Supporting information. [file CL2-20-e1389-s002.docx]

**Appendix B - Screening**

**Stage 1 - Screening based on title and abstract:**

1. Is this an intervention study? (**S**tudy type is exclusion reason)

Yes – include

Uncertain – include

No – stop here and exclude

Question guidance: This review will only include intervention studies. Reviews, meta-analyses, theoretical articles, commentaries, etc. will be excluded.

2. Is the study about Strategy Instructions? (**I**ntervention exclusion reason)

Yes – include

Uncertain – include

No – stop here and exclude

Question guidance: The Strategy Instructions or interventions based on the concept of strategy instruction should include specific use of strategies that involve students planning, writing, revising, and/or editing texts.

3. Is the study about writing performance? (**O**)

Yes – include

Uncertain – include

No – stop here and exclude

Question guidance: The intervention should focus on writing performance as an outcome.

4. Are the participants in the intervention students attending regular, private, or public, schools in grades 7-12 (age 12-19)? (**P**articipant exclusion reason).

Yes – include

Uncertain – include

No – stop here and exclude

Question guidance:

Typical classes include all students attending class. Furthermore, studies of preschool, other early childhood interventions, primary school or secondary school should be excluded. Studies of interventions in tertiary education, such as universities, colleges, technical training institutes, community colleges, nursing schools, research laboratories, centers of excellence, and distance learning centers should also be excluded.

5. Did the intervention take place in school during the regular school year? (**I)**

Yes – include

Uncertain – include

No – stop here and exclude

Question guidance: The intervention should be performed during the regular school year and in school(s), with schools being a stakeholder in the intervention. Interventions performed during e.g. summer or winter breaks should be excluded. If one part of the intervention is performed in school, and another outside of school, the intervention should be included.

6. Is the study a primary impact study reporting quantitative outcomes published in or after 1992?

Yes – include

Uncertain – include

No – stop here and exclude

Question guidance: The study should be primary research, reviews should be excluded. The study should be published in or after the year 1992 to be included.

**Stage 2 - Screening based on full text:**

Second level screening based on full text: Repeat, if necessary, questions 1 – 6 based on full text. Exclude the study if the answer is ‘No’ to one or more of these questions; otherwise, continue with questions 5-7 below.

Exclude the study if the answer to one or more of these three questions is ‘No’. Any remaining uncertainty or disagreement of eligibility will be resolved by the review authors.

7. Is the intervention teacher-delivered in the regular classroom setting?

Yes – include

Uncertain – include

No – stop here and exclude

Question guidance: The scope of this review is focused on teacher-delivered strategy instructions in the regular classroom setting. Interventions in small groups (e.g., special education) are excluded.

8. Does the study report outcomes of story quality, story elements or components, and/ or length and word count?

Yes – include

Uncertain – include

No – stop here and exclude

Question guidance: At least one of the core outcome variables should be measured in the study.

9. Is the study a RCT, Cluster RCT or QES with a control or a comparison group?

Yes – include

Uncertain – include

No – stop here and exclude

Question guidance: Include randomized controlled trials (RCT) and cluster randomized controlled trials. We will also include quasi-experimental designs that use both control groups and pretests. Controls should be carefully matched (e.g, same-year students, same or similar school) with demonstrated baseline equivalence. Single group pre-post comparisons are excluded as well as studies that compare with norm data or similar types of statistical controls. The comparison/control group will be teaching as usual, which does not include strategy instructions or another subtype of strategy instruction.
