# Supplementary material for: Protocol: Strategy instruction for improving short‐ and long‐term writing performance on secondary and upper‐secondary students: A systematic review
Source: Campbell Syst Rev. 2024 Mar 3;20(2):e1389. doi: 10.1002/cl2.1389 (PMC10909389; doi:10.1002/cl2.1389)
Supplement: Supplementary file 3 — Supporting information. [file CL2-20-e1389-s004.docx]

Appendix C - Code book

**1. Report characteristics**

1.1. Authors

1.2. Publishing status

1.3. Publication year

1.4. Type of publication

**2. Study characteristics**

2.1. Study design (Clustered-RCT, Clustered-QEs)

2.2 Number of clusters (classes / schools)

2.3 Estimate of the intracluster (Reported ICC / NA)

**3. Participant characteristics**

3.1. Specify the target group of the intervention: all students or the subgroup of struggling writers. (some studies will be coded twice)

3.3. Age distribution (min, max, mean)

3.4. Grade distribution (min, max, mean)

3.5. Ethnicity/Cultural/Language background (second language)

3.6 Country (what country was the study conducted in)

3.7 Gender distribution

**4. Intervention characteristics**

4.1. Name of intervention (Self-regulated strategy Instruction or CRSI)

4.2. Instructional methods

4.2.1. Describe the instruction methods used in the intervention (eg. numbers of steps) and any differences between treatment and control groups regarding these methods. State explicitly if there are no differences.

4.3. Describe the content domain targeted by the intervention (genre etc.)

4.4. How is the intervention delivered?

4.4.2. Intervention implementer

4.4.3. Is the implementer trained?

4.6. Duration of intervention in weeks (intended, received)

4.7. Frequency of intervention in sessions (intended, received)

4.8. Intended intensity of intervention in hours per week (intended, received)

4.9. Was the implementation of the program monitored by the author/researcher or program personnel to assess whether it was delivered as intended? (Yes/No/NA)

**5. Control/comparison characteristics**

5.1. What is the nature of the control/comparison condition?

- Controls do not receive any intervention/treatment/service (teaching as usual)
- Comparison intervention (if yes, questions regarding participant characteristics and intervention characteristics should be answered for all treatments)

**6. Outcome measurement**

6.1. Measurement timing (pre-post, follow up)

6.2. Name of standardized test (if applicable repeat for all outcomes)

6.3. Who performs the tests?

**7. Sample size**

7.1. Sample size used in analysis for outcome measurement (repeat for all

outcomes and groups)

**8. Outcomes**

8.1. Outcome (repeat for all measurements)

- Continuous measure
- Score type (1 - high score is desirable / 0 - Low score is desirable)
- Standard deviation (incl which groups the standard deviation is sourced from)
- Estimation method (e.g. raw means, adjusted means, regression adjusted etc)
