# Supplementary material for: Protocol: Strategy instruction for improving short‐ and long‐term writing performance on secondary and upper‐secondary students: A systematic review
Source: Campbell Syst Rev. 2024 Mar 3;20(2):e1389. doi: 10.1002/cl2.1389 (PMC10909389; doi:10.1002/cl2.1389)
Supplement: Supplementary file 4 — Supporting information. [file CL2-20-e1389-s003.docx]

APPENDIX D - R CODE

#Code template for the planned analysis.

#For brevity we only show the RCT data for all students.

#Exact same code but different datasets for the other three groups:

#quasi-experimental all students, RCT struggling students and quasi struggling students)

#Loading the libraries

library(metafor)

library(tidyverse)

#Import dataset

#Make sure the data file is in the directory folder of the project

rct <- read_csv("rct_all.csv")

# Apply correction for clusters

#View the data

View(rct)

#Save dataset for all studies including high rob

rct_rob <- rct

View(rct_rob)

#Select only low or moderate bias studies for main analysis.

rct <- filter(rct, rob =! 'high')

View(rct)

#Calculate standardized mean difference and variance for the meta-analysis

effects <- escalc(measure="SMD", m1i=Mean.2.A, m2i=Mean.1.A, sd1i=SD.2.A,sd2i=SD.1.A, n1i=N.2.A, n2i=N.1.A, data=rct)

View(effects)

#Alternative list of effects that includes high ROB studies.

effects_rob <- escalc(measure="SMD", m1i=Mean.2.A, m2i=Mean.1.A, sd1i=SD.2.A,sd2i=SD.1.A, n1i=N.2.A, n2i=N.1.A, data=rct_rob)

#Random effects meta-analysis

res <- rma(yi=yi,vi=vi,data=effects)

res

forest(res, slab = effects$Study)

#####Sensitivity analyses#####

#Leave one out sensitivity analysis

leave1out(res)

#Repeat main analyses with the high ROB incuded.

res_rob <- rma(yi=yi,vi=vi,data=effects_rob)

#####Publication bias####

#standard funnel plot

funnel(res)

#enhanced funnel to check for publication bias

funnel(res, level=c(90, 95, 99), shade=c("white", "gray", "darkgray"), refline=0)

#Below will only be used if K > 10.

#Eggers test for assymetry fo the funnel plot

regtest(res)

#PET and PET-PEESSE

#First run PET

pet <- rma(yi = yi, vi = vi, mods = ~ sqrt(vi), data = effects)

#Then run PEESE.

#Use PEESE estimation if effect size from PET statistically significant.

peese <- rma(yi = yi, vi = vi, mods = ~ vi, data = effects)

#3-PSM selection model based on 5 % alpha level.

#These one estimates the selection based on the data.

#It is very noisy at k = < 20.

selmodel(res, type = "stepfun", steps = 0.025)

# 3-PSM selection model based on 10 % alpha level.

# selmodel(res, type = "stepfun", steps = 0.05)
